# Locally-Referenced Ultrasonic – LPS for Localization and Navigation

**DOI:** 10.3390/s141121750

**Published:** 2014-11-18

**Authors:** David Gualda, Jesús Ureña, Juan C. García, Alejandro Lindo

**Affiliations:** Electronics Department, University of Alcalá de Henares, Escuela Politécnica. Ctra. Madrid-Barcelona, Km. 33,600, 28871 Alcalá de Henares, Spain; E-Mails: urena@depeca.uah.es (J.U.); jcarlos@depeca.uah.es (J.C.G.); alejandro.lindo@depeca.uah.es (A.L.)

**Keywords:** ultrasonic local positioning systems, globally-referenced LPS, locally-referenced LPS, indoor navigation, mobile robot, data fusion

## Abstract

This paper presents a flexible deployment of ultrasonic position sensors and a novel positioning algorithm suitable for the navigation of mobile robots (MRs) in extensive indoor environments. Our proposal uses several independently-referenced local positioning systems (LPS), which means that each one of them operates within its own local reference system. In a typical layout, an indoor extensive area can be covered using just a reduced set of globally-referenced LPS (GRLPS), whose beacon positions are known to the global reference system, while the rest of the space can be covered using locally-referenced LPSs (LRLPS) that can be distributed arbitrarily. The number of LRLPS and their position can be also changed at any moment. The algorithm is composed of several Bayesian filters running in parallel, so that when an MR is under the GRLPS coverage area, its position is updated by a global filter, whereas when the MR is inside the LRLPS area, its position is updated using position increments within a local filter. The navigation algorithm has been tested by simulation and with actual data obtained using a real set of ultrasonic LPSs.

## 1. Introduction

The estimation of the position and navigation tasks of a mobile robot (MR) in indoor environments has been an important research topic during the last few years. Some systems try to replicate indoors the GPS behavior and performances outdoors. The most popular technologies used in order to implement an indoor positioning system are Ultrasound (US) [1–4] and Radiofrequency (RF) [5–7].

Usually, these technologies are used to build local position systems (LPS) [8–10] composed of a set of beacons, placed on known fixed positions that emit a signal (RF, US), which is captured by a mobile receptor in order to estimate its position. Using some kind of distance-dependent information included within the beacons' signals (*i.e.*, time of flight, signal intensity), the receptor can estimate its position. If the recovered information is an absolute distance to each beacon, the estimation of the position can be obtained by a spherical trilateration [11]. The drawback of the spherical trilateration is that it is necessary to synchronize in time beacons and receivers. Alternatively, using the difference of distances to a reference beacon, the estimation of the position can be computed using a hyperbolic trilateration algorithm [12]. However, in such case of hyperbolic trilateration, the minimum set of beacons needed to estimate the receiver's position increases.

In the case of MR navigation, the most used positioning technique is odometry, based on the information provided by the encoders linked with their movement axes. Nevertheless, odometry is a relative positioning system whose estimations have an error that gets higher with time and that can also reflect impulsive error behavior related with skids or some other traction defects. Using process filtering, the odometry errors can be lowered to some grade. The most commonly used algorithms are based on Bayesian theory, like the unscented and extended Kalman filter (EKF, UKF) [13,14], the H-infinity [15] and the particle filter [16].

The best way to prevent heavy odometry errors is to fuse this source of information with complementary absolute positioning systems, like GPS outdoors. However, indoors, GPS cannot be used because of degradations in the reception from satellites, and that is why LPS are needed in those environments.

In extensive areas (for instance, inside large buildings), the deployed LPSs should cover most of the area, using as many additional LPS around as required by the degree of precision needed in the positioning task [10]. Ultrasonic beacons, for instance, give very good results when measuring distances by time of flight techniques (TOF), but the reduced range forces one to install several of these systems to cover large areas. Moreover, each LPS needs to be calibrated at their position after being deployed and before beginning the localization tasks. Therefore, the deployment of such systems is complicated when there are dynamic changes or a fast installation is required, for instance in emergencies or similar situations. If some or all of the LPSs in an extensive area could be installed without a previous calibration stage, many of those drawbacks would be avoided.

This paper presents a novel algorithm that allows one to navigate an MR in extensive areas using a set of LPSs, most of them without calibration. Section 2 describes the scenario and the statement of the problem. Section 3 introduces the proposed algorithm in both the spherical and the hyperbolic cases. Section 4 shows some simulated results. Section 5 presents real results. Section 6 describes the possibility to adapt the system for human tracking, and finally, Section 7 discusses some conclusions derived from the proposal.

## 2. Statement of the Problem and Definitions

**Figure 1.**
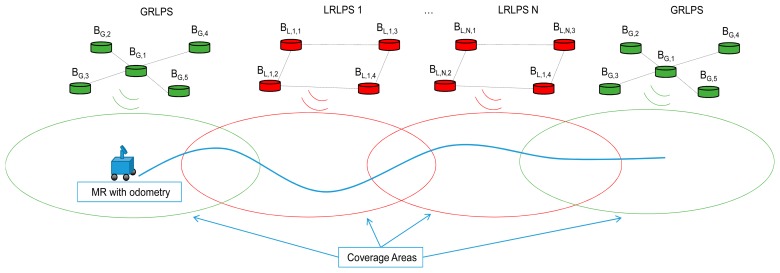
Particular case of globally-referenced LPS (GRLPS) and locally-referenced LPS (LRLPS) structures, with five and four beacons, respectively. MR, mobile robot.

The test scenario is an extended indoor area where it is necessary to locate an MR. This large area is covered using a set of calibrated and uncalibrated LPSs (Figure 1). A calibrated LPS is the one whose beacons have been referenced to a global system, for instance after a calibration procedure; we will denote it as globally-referenced LPS (GRLPS). Normally, a GRLPS will be placed in strategic waypoints, like, for instance, the main entrances to a section in a building. The uncalibrated LPS will be identified as locally-referenced LPS (LRLPS), because only the relative positions of the beacons inside each LRLPS are known, and they have been determined prior to the deployment; they only depend on the geometry of the LPS and not on the place in which it is installed. LRLPSs can be placed all around the movement area, and their number and positions can be changed dynamically (at any time).

**Figure 2.**
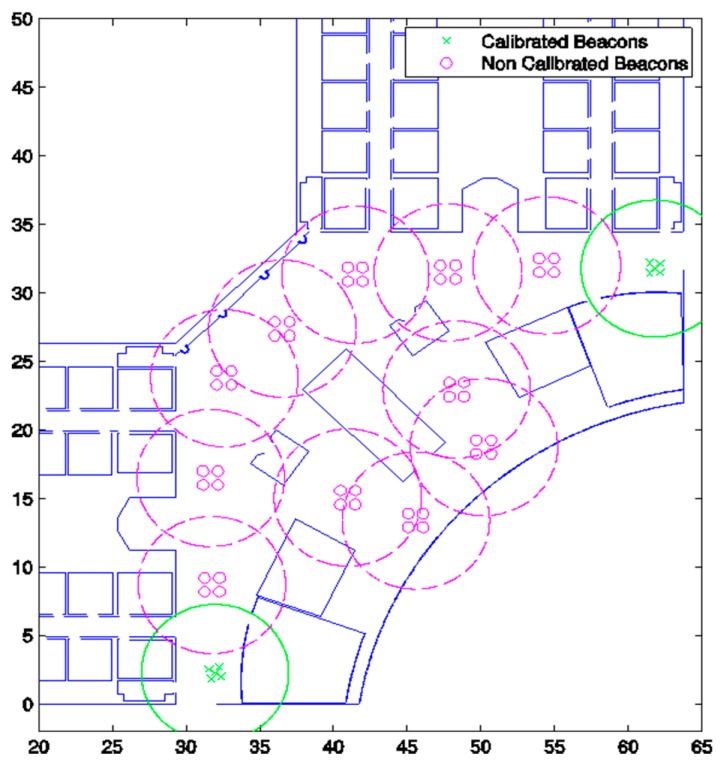
Simulated workspace based on the real plan of a building.

Each LPS has to be composed of at least three beacons in the spherical case or four beacons in the hyperbolic one, in order to have enough distance measurements to apply the algorithms. Figure 2 shows a simulated scenario, where all beacons are placed on the ceiling, including GRLPSs, LRLPSs and their coverage areas on the ground. In this particular case, the GRPLS is composed of five beacons and the LRLPS of four beacons (so the GRLPS has more redundancy). Because of the performances achieved with the actual LPS used in real tests [9], we will suppose that for all of the LPSs, the coverage area is a circle on the ground with a radius of five meters.

The structure of each LPS must be known. In this case, we have used four beacons for each non-calibrated LPS structure (the LRLPS) composing a square with a side of 1 m; and five beacons, one more in the center of the square, for each calibrated LPS structure (the GRLPS). Some kind of overlapping between the LRLPSs' coverage area is recommended in order to reduce the positioning error of a mobile robot while navigating in the environment. If the MR moves outside the LPS coverage area (either a GR or LR LPS), it will only estimate its position using the odometry, so the error will increase accumulatively.

In order to make the paper more self-contained and to understand the proposed algorithm, some notations are included in this section:

**Table:** 

**X̂**_G_*_,k_*:	Global state vector at instant *k*. This vector is composed of the global coordinates of the MR and its orientation. G indicates that the reference is global.
**X̂**_L,_*_n_*_,_*_k_*:	Local state vector at instant *k*. This vector is composed of the local coordinates of the MR and its orientation. L indicates that the reference is local, and *n* is the specific LRLPS.
**X̂̄**_G_*_,k_*	*A priori* global state vector at instant *k*.
**X̂̄**_L,_*_n_*_,_*_k_*:	*A priori n*-local state vector at instant *k*.
*d_pG,k,bG,m_*:	Real distance measured between the global position of the MR at instant *k* and the *m*-th global beacon of GRLPS.
*d̂_p_*_G_*_,k,b_*_G_*_,m_*:	Estimated distance between the global position of the MR at instant *k* and the *m*-th global beacon of GRLPS.
*d_p_*_L_*_,k,b_*_L_*_,m_*:	Real distance measured between the local position of the MR at instant *k* and the *m*-th local beacon of the *n*-LRLPS.
*d̂_p_*_L_*_,k,b_*_L_*_,m_*:	Estimated distance between the local position of the MR at instant *k* and the *m*-th local beacon of the *n*-LRLPS.
Δ*d_p_*_G_*_,k,b_*_G_*_,m_*:	Real difference of distances measured between beacon *m* and beacon *1* of GRLPS to the global position of the MR at instant *k*.
Δ*d̂_p_*_G_*_,k,b_*_G_*_,m_*:	Estimated difference of distances between beacon *m* and beacon *1* of GRLPS to the global position of the MR at instant *k*.
Δ*d_p_*_L_*_,k,b_*_L_*_,m_*:	Real difference of distances measured between beacon *m* and beacon *1* of the *n*-LRLPS to the global position of the MR at instant *k*.
Δ*d̂_p_*_L_*_,k,b_*_L_*_,m_*:	Estimated difference of distanced between beacon *m* and beacon *1* of the *n*-LRLPS to the global position of the MR at instant *k*.
**A**_G,_*_k_*:	Global transition matrix of the dynamic model.
**A**_L,_*_n_*_,_*_k_*:	*n*-local transition matrix of the dynamic model.
*h*(**X̂̄**_G,_*_k_*):	Global matrix observations obtained by the *a priori* information.
*h*(**X̂̄**_L,_*_n_*_,_*_m_*):	*n*-local matrix observations obtained by the n-local *a priori* information.
**H**_G,_*_k_*:	Global measurement model matrix.
**H**_L,_*_n_*_,_*_k_*:	*n*-local measurement model matrix.
**P**_G,_*_k_*:	Global covariance matrix
**P**_L,_*_n_*_,_*_k_*:	*n*-local covariance matrix.
**Q**_G,_*_k_*:	*Global process noise covariance matrix.*
**Q**_L,_*_n_*_,_*_k_*:	*n-local process noise covariance matrix.*
**R**_G_:	*Global measure noise covariance matrix.*
**R**_L,_*_n_*:	*n*-local measure noise covariance matrix.
$\sigma_{w_{G}}^{2}$:	Global measure variance (variance of distance in spherical case or difference of distances in hyperbolic one).
$\sigma_{w_{L,n}}^{2}$:	*n*-local measure variance.

## 3. Proposed Algorithm

The pseudocode of the proposed algorithm is presented in Algorithm 1, and it can be applied for both the spherical and the hyperbolic cases. Basically, this pseudocode points out which state vector (local or global) is updated depending on the measurements performed by the MR according to its localization and the coverage area of the different LPSs. The process for the main lines of this pseudocode is explained with more detail in the following paragraphs.

The algorithm estimates the 2D location and the orientation of a mobile node inside the global coordinates system. The estimated position values for the actual instant *k* are described by the global state vector (1):

(1) $${\overset{⌢}{\text{X}}}_{\text{G},k}={\left[\begin{array}{ccc} {\overset{⌢}{x}}_{\text{G},k} & {\overset{⌢}{y}}_{\text{G},k} & {\overset{⌢}{\theta }}_{\text{G},k} \end{array}\right]}^{T}$$

When the mobile node is located inside the coverage area of a LRLPS, another local state vector (2) is defined additionally to the global vector:

(2) $${\overset{⌢}{\text{X}}}_{\text{L},n,k}={\left[\begin{array}{ccc} {\overset{⌢}{x}}_{\text{L},n,k} & {\overset{⌢}{y}}_{\text{L},n,k} & {\overset{⌢}{\theta }}_{\text{L},n,k} \end{array}\right]}^{T}$$

where *n* = 1, 2, …, *N* is the specific LRLPS.

In order to have always a globally referenced position and orientation of the MR, let us suppose that the first iteration of this algorithm will be computed when the MR is inside the coverage area of a GRLPS. This is a convenient condition and is also quite easy to accomplish, because GRLPSs have been specified to be installed on the entry points of the main building sections' areas of interest.

**Table:** 

**Algorithm 1.** Pseudocode of the proposed algorithm.	
1:	**while** the MR is navigating … **do**
2:	**if** (GRLPS coverage exists) **then**
3:	**if** (first iteration of GRLPS) **then**
4:	Global vector initialization
5:	Global covariance matrix initialization
6:	**else**
7:	Global state vector update using EKF
8:	Global covariance matrix update
9:	**end if**
10:	**else if** (LRLPS coverage exists) **then**
11:	**for all** LRLPS with coverage **do**
12:	**if** (first iteration of LRLPS(n)) **then**
13:	Local vector initialization
14:	Local covariance matrix update
15:	**else**
16:	Local state vector update using EKF
17:	Local covariance matrix update
18:	**end if**
19:	**end for**
20:	Global state vector update using the increment of distance and angle between *k* and *k* – 1 iteration of the local state vector that has the least value of its covariance matrix
21:	**else**
22:	Global state vector update using only odometry
23:	Global covariance matrix update
24:	**end if**
25:	**end while**

### 3.1. Global Initialization for the GRLPS Area (Lines 4 and 5 in Algorithm 1)

When the MR goes into a GRLPS area for the first time, the global state vector (3) is obtained using a Gauss–Newton minimization algorithm in order to minimize the Equations (4) or (5) depending on the case (spherical or hyperbolic):

(3) $${\overset{⌢}{\text{X}}}_{\text{G},0}={\left[\begin{array}{ccc} {\overset{⌢}{x}}_{\text{G},0} & {\overset{⌢}{y}}_{\text{G},0} & {\overset{⌢}{\theta }}_{\text{G},0} \end{array}\right]}^{T}$$

(4) $${\left[\begin{array}{cc} {\overset{⌢}{x}}_{\text{G},0} & {\overset{⌢}{y}}_{\text{G},0} \end{array}\right]}^{T}=\arg\min\sum_{m=1}^{M}\left(\left|d_{p_{\text{G},0},b_{\text{G},m}}-{\hat{d}}_{p_{\text{G},0},b_{\text{G},m}}\right|\right)$$

(5) $${\left[\begin{array}{cc} {\overset{⌢}{x}}_{\text{G},0} & {\overset{⌢}{y}}_{\text{G},0} \end{array}\right]}^{T}=\arg\min\sum_{m=2}^{M}\left(\left|\Delta d_{p_{\text{G},0},b_{\text{G},m}}-\Delta {\hat{d}}_{p_{\text{G},0},b_{\text{G},m}}\right|\right)$$

where *m* = 1, 2, …, *M* represents each specific beacon of this GRLPS. Note that in the hyperbolic case, *m* = 2, 3, …, *M*, because the first beacon is the reference one.

In the case of spherical trilateration, the estimated distance between the MR to each beacon for that specific GRLPS is used as defined in Equation (6):

(6) $${\hat{d}}_{p_{\text{G},0},b_{\text{G},m}}=\sqrt{{\left({\hat{x}}_{\text{G},0}-xb_{\text{G},m}\right)}^{2}+{\left({\hat{y}}_{\text{G},0}-yb_{\text{G},m}\right)}^{2}+{\left(z_{\text{MR}}-zb_{\text{G},m}\right)}^{2}}$$

where *z*_MR_ is the height of the MR.

For the hyperbolic trilateration, the differences of distances are used, as defined in Equation (7):

(7) $$\begin{array}{c} \Delta {\hat{d}}_{p_{\text{G},0},b_{\text{G},m}}=\sqrt{{\left({\hat{x}}_{\text{G},0}-xb_{\text{G},m}\right)}^{2}+{\left({\hat{y}}_{\text{G},0}-yb_{\text{G},m}\right)}^{2}+{\left(z_{\text{MR}}-zb_{\text{G},m}\right)}^{2}}\\ -\sqrt{{\left({\hat{x}}_{\text{G},0}-xb_{\text{G},1}\right)}^{2}+{\left({\hat{y}}_{\text{G},0}-yb_{\text{G},1}\right)}^{2}+{\left(z_{\text{MR}}-zb_{\text{G},1}\right)}^{2}} \end{array}$$

### 3.2. Global Navigation inside a GRLPS Area (Lines 7 and 8 in Algorithm 1)

For the rest of iterations, when the robot is navigating inside a GRLPS area, the global state vector and its covariance matrix are updated following the EKF structure:

Prediction stage:

(8) $${\overset{⌢}{\text{X}}}_{\text{G},k}^{-}=f\left({\overset{⌢}{\text{X}}}_{\text{G},k-1}^{-},{\text{u}}_{k}\right)$$

(9) $${\text{P}}_{\text{G},k}^{-}={\text{A}}_{\text{G},k}\cdot {\text{P}}_{\text{G},k-1}\cdot {\text{A}}_{\text{G},k}^{T}+{\text{Q}}_{\text{G}}$$

Update stage

(10) $$\text{K}={\text{P}}_{\text{G},k}^{-}\cdot {\text{H}}_{\text{G},k}^{T}\cdot {\left[{\text{H}}_{\text{G},k}\cdot {\text{P}}_{\text{G},k}^{-}\cdot {\text{H}}_{\text{G},k}^{T}+{\text{H}}_{\text{G},k}^{T}+{\text{R}}_{\text{G}}\right]}^{-1}$$

(11) $${\overset{⌢}{\text{X}}}_{\text{G},k}^{-}={\overset{⌢}{\text{X}}}_{\text{G},k}+\text{K}\cdot \left({\text{Z}}_{\text{G},k}-h\left({\overset{⌢}{\text{X}}}_{\text{G},k}^{-}\right)\right)$$

(12) $${\text{P}}_{\text{G},k}=\left(\text{I}-\text{K}\cdot {\text{H}}_{\text{G},k}\right)\cdot {\text{P}}_{\text{G},k}^{-}$$

Let us suppose that Δ*d_odo_* is the increment of distance and Δθ*_odo_* is the angular increment extracted from the MR odometry. The *a priori* estimation of the global state vector is defined by Equation (13), where we have used such odometry information to estimate this state.

(13) $${\overset{⌢}{\text{X}}}_{\text{G},k}^{-}=\left[\begin{array}{c} {\hat{x}}_{\text{G},k-1}+\Delta d_{\text{odo}}\cdot \cos\left({\hat{\theta }}_{\text{G},k-1}+\Delta \theta_{\text{odo}}\right)\\ {\hat{y}}_{\text{G},k-1}+\Delta d_{\text{odo}}\cdot \sin\left({\hat{\theta }}_{\text{G},k-1}+\Delta \theta_{\text{odo}}\right)\\ {\hat{\theta }}_{\text{G},k-1}+\Delta \theta_{\text{odo}} \end{array}\right]$$

The global transition matrix of the dynamic model is defined by Equation (14):

(14) $${\text{A}}_{\text{G},k}\left[\begin{array}{c} \frac{\partial f\left({\hat{\text{X}}}_{\text{G},k}^{-},{\text{u}}_{k}\right)}{\partial {\hat{x}}_{\text{G},k}}\\ \frac{\partial f\left({\hat{\text{X}}}_{\text{G},k}^{-},{\text{u}}_{k}\right)}{\partial {\hat{y}}_{\text{G},k}}\\ \frac{\partial f\left({\hat{\text{X}}}_{\text{G},k}^{-},{\text{u}}_{k}\right)}{\partial {\hat{\theta }}_{\text{G},k}} \end{array}\right]=\left[\begin{array}{ccc} 1 & 0 & -\Delta d_{\text{odo}}\cdot \sin\left({\hat{\theta }}_{\text{G},k-1}+\Delta \theta_{\text{odo}}\right)\\ 0 & 1 & \Delta d_{\text{odo}}\cdot \cos\left({\hat{\theta }}_{\text{G},k-1}+\Delta \theta_{\text{odo}}\right)\\ 0 & 0 & 1 \end{array}\right]$$

The global covariance matrix is obtained by Equation (15):

(15) $${\text{P}}_{\text{G},k}=\left[\begin{array}{ccc} \sigma_{x_{\text{G}}}^{2} & \sigma_{x_{\text{G}},y_{\text{G}}}^{2} & \sigma_{x_{\text{G}},\theta_{\text{G}}}^{2}\\ \sigma_{y_{\text{G}},x_{\text{G}}}^{2} & \sigma_{y_{\text{G}}}^{2} & \sigma_{y_{\text{G}},\theta_{\text{G}}}^{2}\\ \sigma_{\theta_{\text{G}},x_{\text{G}}}^{2} & \sigma_{\theta_{\text{G}},y_{\text{G}}}^{2} & \sigma_{\theta_{\text{G}}}^{2} \end{array}\right]$$

and the process noise is modeled by the diagonal matrix (16).

(16) $${\text{Q}}_{\text{G}}=\left[\begin{array}{ccc} \sigma_{v_{\text{G},x}}^{2} & 0 & 0\\ 0 & \sigma_{v_{\text{G},y}}^{2} & 0\\ 0 & 0 & \sigma_{v_{\text{G},\theta }}^{2} \end{array}\right]$$

where $\sigma_{v_{\text{G},x}}^{2}$, $\sigma_{v_{\text{G},y}}^{2}$ and $\sigma_{v_{\text{G},\theta }}^{2}$ are the variances related to each value of the global state vector.

The global matrix observations obtained by the *a priori* information are shown in Equation (17) for the spherical case. Its derivative represents the global measurement model matrix and is defined in Equation (18):

(17) $$h\left({\hat{\text{X}}}_{\text{G},k}^{-}\right)=\left[\begin{array}{c} h_{1}\left({\hat{\text{X}}}_{\text{G},k}^{-}\right)\\ \vdots \\ h_{M}\left({\hat{\text{X}}}_{\text{G},k}^{-}\right) \end{array}\right]=\left[\begin{array}{c} \sqrt{{\left({\hat{x}}_{\text{G},k}^{-}-xb_{\text{G},1\;}\right)}^{2}+{\left({\hat{y}}_{\text{G},k}^{-}-yb_{\text{G},1\;}\right)}^{2}+{\left(z_{\text{MR}\;}-zb_{\text{G},1\;}\right)}^{2}}\\ \vdots \\ \sqrt{{\left({\hat{x}}_{\text{G},k}^{-}-xb_{\text{G},1\;}\right)}^{2}+{\left({\hat{y}}_{\text{G},k}^{-}-yb_{\text{G},1\;}\right)}^{2}+{\left(z_{\text{MR}\;}-zb_{\text{G},m\;}\right)}^{2}} \end{array}\right]$$

(18) $${\text{H}}_{\text{G},k}=\left[\begin{array}{c} \frac{\partial h_{1}\left({\hat{\text{X}}}_{\text{G},k}^{-}\right)}{\partial \left({\hat{\text{X}}}_{\text{G},k}^{-}\right)}\\ \vdots \\ \frac{\partial h_{M}\left({\hat{\text{X}}}_{\text{G},k}^{-}\right)}{\partial \left({\hat{\text{X}}}_{\text{G},k}^{-}\right)} \end{array}\right]=\left[\begin{array}{ccc} \frac{\partial h_{1}\left({\hat{\text{X}}}_{\text{G},k}^{-}\right)}{\partial \left({\hat{x}}_{\text{G},k}^{-}\right)} & \frac{\partial h_{1}\left({\hat{\text{X}}}_{\text{G},k}^{-}\right)}{\partial \left({\hat{y}}_{\text{G},k}^{-}\right)} & \frac{\partial h_{1}\left({\hat{\text{X}}}_{\text{G},k}^{-}\right)}{\partial \left({\hat{\theta }}_{\text{G},k}^{-}\right)}\\ \vdots & \vdots & \vdots \\ \frac{\partial h_{M}\left({\hat{\text{X}}}_{\text{G},k}^{-}\right)}{\partial \left({\hat{x}}_{\text{G},k}^{-}\right)} & \frac{\partial h_{M}\left({\hat{\text{X}}}_{\text{G},k}^{-}\right)}{\partial \left({\hat{y}}_{\text{G},k}^{-}\right)} & \frac{\partial h_{M}\left({\hat{\text{X}}}_{\text{G},k}^{-}\right)}{\partial \left({\hat{\theta }}_{\text{G},k}^{-}\right)} \end{array}\right]$$

Regarding the hyperbolic case, the global matrix observation is given by Equation (19):

(19) $$h\left({\hat{\text{X}}}_{\text{G},k}^{-}\right)=\left[\begin{array}{c} h_{1}\left({\hat{\text{X}}}_{\text{G},k}^{-}\right)\\ \vdots \\ h_{M}\left({\hat{\text{X}}}_{\text{G},k}^{-}\right) \end{array}\right]=\left[\begin{array}{c} \sqrt{{\left({\hat{x}}_{\text{G},k}^{-}-xb_{\text{G},2\;}\right)}^{2}+{\left({\hat{y}}_{\text{G},k}^{-}-yb_{\text{G},2\;}\right)}^{2}+{\left(z_{\text{MR}\;}-zb_{\text{G},2\;}\right)}^{2}}\\ -\sqrt{{\left({\hat{x}}_{\text{G},k}^{-}-xb_{\text{G},1\;}\right)}^{2}+{\left({\hat{y}}_{\text{G},k}^{-}-yb_{\text{G},1\;}\right)}^{2}+{\left(z_{\text{MR}\;}-zb_{\text{G},1\;}\right)}^{2}}\\ \vdots \\ \sqrt{{\left({\hat{x}}_{\text{G},k}^{-}-xb_{\text{G},M\;}\right)}^{2}+{\left({\hat{y}}_{\text{G},k}^{-}-yb_{\text{G},M\;}\right)}^{2}+{\left(z_{\text{MR}\;}-zb_{\text{G},M\;}\right)}^{2}}\\ -\sqrt{{\left({\hat{x}}_{\text{G},k}^{-}-xb_{\text{G},1\;}\right)}^{2}+{\left({\hat{y}}_{\text{G},k}^{-}-yb_{\text{G},1\;}\right)}^{2}+{\left(z_{\text{MR}\;}-zb_{\text{G},1\;}\right)}^{2}} \end{array}\right]$$

For the spherical case, as the distance measurements are directly used, we can assume that the noise for each measurement is Gaussian and uncorrelated, so **R_G_** is a diagonal matrix as defined in Equation (20), whose diagonal elements are the variance in the distance measurements:

(20) $${\text{R}}_{\text{G}}=\left[\begin{array}{ccc} \sigma_{w_{\text{G}}}^{2} & 0 & 0\\ 0 & \ddots & 0\\ 0 & 0 & \sigma_{w_{\text{G}}}^{2} \end{array}\right]$$

For the hyperbolic case, we also assume that the noise is Gaussian, but now, as differences of distances are used, the noise is correlated [17] and **R_G_** is the matrix shown in Equation (21):

(21) $${\text{R}}_{\text{G}}=\left[\begin{array}{ccc} \sigma_{w_{\text{G}}}^{2} & 0.5\cdot \sigma_{w_{\text{G}}}^{2} & 0.5\cdot \sigma_{w_{\text{G}}}^{2}\\ 0.5\cdot \sigma_{w_{\text{G}}}^{2} & \ddots & 0.5\cdot \sigma_{w_{\text{G}}}^{2}\\ 0.5\cdot \sigma_{w_{\text{G}}}^{2} & 0.5\cdot \sigma_{w_{\text{G}}}^{2} & \sigma_{w_{\text{G}}}^{2} \end{array}\right]$$

### 3.3. Local Initialization for the LRLPS Area (Lines 13 and 14 in Algorithm 1)

When the MR moves into an LRLPS area, the first iteration of the *n*-local vector (22) is obtained using the same procedure as for the GRLPS case. The aim is to minimize the Equations (23) or (24) depending on the case, spherical or hyperbolic, respectively:

(22) $${\overset{⌢}{\text{X}}}_{\text{L},n,0}={\left[\begin{array}{ccc} {\hat{x}}_{\text{L},n,0} & {\hat{y}}_{\text{L},n,0} & {\hat{\theta }}_{\text{L},n,0} \end{array}\right]}^{T}$$

(23) $${\left[\begin{array}{cc} {\overset{⌢}{x}}_{\text{L},\text{n},0} & {\overset{⌢}{y}}_{\text{L},\text{n},0} \end{array}\right]}^{T}=\arg\min\sum_{m=1}^{M}\left(\left|d_{p_{\text{L},\text{n},0},b_{\text{L},\text{n},m}}-{\hat{d}}_{p_{\text{L},\text{n},0},b_{\text{L},\text{n},m}}\right|\right)$$

(24) $${\left[\begin{array}{cc} {\overset{⌢}{x}}_{\text{L},\text{n},0} & {\overset{⌢}{y}}_{\text{L},\text{n},0} \end{array}\right]}^{T}=\arg\min\sum_{m=2}^{M}\left(\left|\Delta d_{p_{\text{L},\text{n},0},b_{\text{L},\text{n},m}}-\Delta {\hat{d}}_{p_{\text{L},\text{n},0},b_{\text{L},\text{n},m}}\right|\right)$$

where *m* = 1, 2, …, *M* represents each beacon of the LRLPS and the distances are calculated by Equations (25) and (26) in each case.

(25) $${\hat{d}}_{p_{\text{L},n,0},b_{\text{L},n,m}}=\sqrt{{\left({\hat{x}}_{\text{L},n,0\;}-xb_{\text{L},n,m\;}\right)}^{2}+{\left({\hat{y}}_{\text{L},n,0\;}-yb_{\text{L},n,m\;}\right)}^{2}+{\left(z_{\text{MR}\;}-zb_{\text{L},n,m\;}\right)}^{2}}$$

(26) $$\begin{array}{c} \Delta {\hat{d}}_{p_{\text{L},n,0},b_{\text{L},n,m}}=\sqrt{{\left({\hat{x}}_{\text{L},n,0\;}-xb_{\text{L},n,m\;}\right)}^{2}+{\left({\hat{y}}_{\text{L},n,0\;}-yb_{\text{L},n,m\;}\right)}^{2}+{\left(z_{\text{MR}\;}-zb_{\text{L},n,m\;}\right)}^{2}}\\ -\sqrt{{\left({\hat{x}}_{\text{L},n,0\;}-xb_{\text{L},n,1\;}\right)}^{2}+{\left({\hat{y}}_{\text{L},n,0\;}-yb_{\text{L},n,1\;}\right)}^{2}+{\left(z_{\text{MR}\;}-zb_{\text{L},n,1\;}\right)}^{2}} \end{array}$$

### 3.4. Local Navigation inside an LRLPS Area (Lines 16 and 17 of Algorithm 1)

If the MR navigates inside a LRLPS area, the local state vector and the covariance matrix are updated with an extended Kalman filter (EKF) particularized to the specific LRLPS and run in parallel to the global one and other LRLPS in the case that the corresponding coverage areas intersect. The Equations are described as follows:

Prediction stage:

(27) $${\overset{⌢}{\text{X}}}_{\text{L},n,k}^{-}=f\left({\overset{⌢}{\text{X}}}_{\text{L},n,k-1},{\text{u}}_{k}\right)$$

(28) $${\text{P}}_{\text{L},n,k}^{-}={\text{A}}_{\text{L},n,k}\cdot {\text{P}}_{\text{L},n,k-1}\cdot {\text{A}}_{\text{L},n,k}^{T}+{\text{Q}}_{\text{L}}$$

Update stage:

(29) $$\text{K}={\text{P}}_{\text{L},n,k}^{-}\cdot {\text{H}}_{\text{L},n,k}^{T}\cdot {\left[{\text{H}}_{\text{L},n,k}\cdot {\text{P}}_{\text{L},n,k}^{-}\cdot {\text{H}}_{\text{L},n,k}^{T}+{\text{H}}_{\text{L},n,k}^{T}+{\text{R}}_{\text{L}}\right]}^{-1}$$

(30) $${\hat{\text{X}}}_{\text{L},n,k}^{-}={\hat{\text{X}}}_{\text{L},n,k}+\text{K}\cdot \left({\text{Z}}_{\text{L},n,k}-h\left({\hat{\text{X}}}_{\text{L},n,k}^{-}\right)\right)$$

(31) $${\text{P}}_{\text{L},n,k}=\left(Ι-\text{K}\cdot {\text{H}}_{\text{L},n,k}\right)\cdot {\text{P}}_{\text{L},n,k}^{-}$$

The *a priori* information of the *n*-local state vector is defined by Equation (32), in which, we have used the odometry information (Δ*d_odo_*, Δθ*_odo_*) in order to estimate this state:

(32) $${\hat{\text{X}}}_{\text{L},n,k}^{-}=\left[\begin{array}{c} {\hat{x}}_{\text{L},n,k-1}+\Delta d_{\text{odo}}\cdot \cos\left({\hat{\theta }}_{\text{L},n,k-1\;}+\Delta \theta_{\text{odo}}\right)\\ {\hat{y}}_{\text{L},n,k-1}+\Delta d_{\text{odo}}\cdot \sin\left({\hat{\theta }}_{\text{L},n,k-1\;}+\Delta \theta_{\text{odo}}\right)\\ {\hat{\theta }}_{\text{L},n,k-1\;}+\Delta \theta_{\text{odo}} \end{array}\right]$$

The Expression (33) is obtained taking the derivative of the matrix (32) with respect to each one of the local state variables:

(33) $${\text{A}}_{\text{L},n,k}=\left[\begin{array}{c} \frac{\partial f\left({\hat{\text{X}}}_{\text{L},n,k}^{-},{\text{u}}_{k}\right)}{\partial {\hat{x}}_{\text{L},n,k\;}}\\ \frac{\partial f\left({\hat{\text{X}}}_{\text{L},n,k}^{-},{\text{u}}_{k}\right)}{\partial {\hat{y}}_{\text{L},n,k\;}}\\ \frac{\partial f\left({\hat{\text{X}}}_{\text{L},n,k}^{-},{\text{u}}_{k}\right)}{\partial {\hat{\theta }}_{\text{L},n,k\;}} \end{array}\right]=\left[\begin{array}{ccc} 1 & 0 & -\Delta d_{\text{odo}}\cdot \sin\left({\hat{\theta }}_{\text{L},n,k-1\;}+\Delta \theta_{\text{odo}}\right)\\ 0 & 1 & \Delta d_{\text{odo}}\cdot \cos\left({\hat{\theta }}_{\text{L},n,k-1\;}+\Delta \theta_{\text{odo}}\right)\\ 0 & 0 & 1 \end{array}\right]$$

The local covariance matrix (34) represents the variance for each local state variable and is updated in every iteration of the local EKF:

(34) $${\text{P}}_{L,n,k}=\left[\begin{array}{ccc} \sigma_{x_{\text{L},n}}^{2} & \sigma_{x_{\text{L},n},y_{\text{L},n}}^{2} & \sigma_{x_{\text{L},n},\theta_{\text{L},n}}^{2}\\ \sigma_{y_{\text{L},n},x_{\text{L},n}}^{2} & \sigma_{y_{\text{L},n}}^{2} & \sigma_{y_{\text{L},n},\theta_{\text{L},n}}^{2}\\ \sigma_{\theta_{\text{L},n},x_{\text{L},n}}^{2} & \sigma_{\theta_{\text{L},n},y_{\text{L},n}}^{2} & \sigma_{\theta_{\text{L},n}}^{2} \end{array}\right]$$

Regarding the process noise, it is modeled by a diagonal matrix (35):

(35) $${\text{Q}}_{\text{L},n}=\left[\begin{array}{ccc} \sigma_{v_{\text{L},n,x}}^{2} & 0 & 0\\ 0 & \sigma_{v_{\text{L},n,y}}^{2} & 0\\ 0 & 0 & \sigma_{v_{\text{L},n,\theta }}^{2} \end{array}\right]$$

The local matrix observations obtained by the *a priori* information is shown in Equation (36) for the spherical case. Its derivative represents the global measurement model matrix and is defined in Equation (37):

(36) $$\begin{array}{c} h\left({\hat{\text{X}}}_{\text{L},n,k}^{-}\right)=\left[\begin{array}{c} h_{1}\left({\hat{\text{X}}}_{\text{L},n,k}^{-}\right)\\ \vdots \\ h_{M}\left({\hat{\text{X}}}_{\text{L},n,k}^{-}\right) \end{array}\right]=\\ =\left[\begin{array}{c} \sqrt{{\left({\hat{x}}_{\text{L},n,k}^{-}-xb_{\text{L},n,1\;}\right)}^{2}+{\left({\hat{y}}_{\text{L},n,k}^{-}-yb_{\text{L},n,1\;}\right)}^{2}+{\left(z_{\text{MR}\;}-zb_{\text{L},n,1\;}\right)}^{2}}\\ \vdots \\ \sqrt{{\left({\hat{x}}_{\text{L},n,k}^{-}-xb_{\text{L},n,1\;}\right)}^{2}+{\left({\hat{y}}_{\text{L},n,k}^{-}-yb_{\text{L},n,1\;}\right)}^{2}+{\left(z_{\text{MR}\;}-zb_{\text{L},n,m\;}\right)}^{2}} \end{array}\right] \end{array}$$

(37) $${\text{H}}_{\text{L},n,k}=\left[\begin{array}{c} \frac{\partial h_{1}\left({\hat{\text{X}}}_{\text{L},n,k}^{-}\right)}{\partial \left({\hat{\text{X}}}_{\text{L},n,k}^{-}\right)}\\ \vdots \\ \frac{\partial h_{M}\left({\hat{\text{X}}}_{\text{L},n,k}^{-}\right)}{\partial \left({\hat{\text{X}}}_{\text{L},n,k}^{-}\right)} \end{array}\right]=\left[\begin{array}{ccc} \frac{\partial h_{1}\left({\hat{\text{X}}}_{\text{L},n,k}^{-}\right)}{\partial \left({\hat{x}}_{\text{L},n,k}^{-}\right)} & \frac{\partial h_{1}\left({\hat{\text{X}}}_{\text{L},n,k}^{-}\right)}{\partial \left({\hat{y}}_{\text{L},n,k}^{-}\right)} & \frac{\partial h_{1}\left({\hat{\text{X}}}_{\text{L},n,k}^{-}\right)}{\partial \left({\hat{\theta }}_{\text{L},n,k}^{-}\right)}\\ \vdots & \vdots & \vdots \\ \frac{\partial h_{M}\left({\hat{\text{X}}}_{\text{L},n,k}^{-}\right)}{\partial \left({\hat{x}}_{\text{L},n,k}^{-}\right)} & \frac{\partial h_{M}\left({\hat{\text{X}}}_{\text{L},n,k}^{-}\right)}{\partial \left({\hat{y}}_{\text{L},n,k}^{-}\right)} & \frac{\partial h_{M}\left({\hat{\text{X}}}_{\text{L},n,k}^{-}\right)}{\partial \left({\hat{\theta }}_{\text{L},n,k}^{-}\right)} \end{array}\right]$$

In the hyperbolic case, the observation matrix is defined by Equation (38):

(38) $$\begin{array}{c} h\left({\hat{\text{X}}}_{\text{L},n,k}^{-}\right)=\left[\begin{array}{c} h_{1}\left({\hat{\text{X}}}_{\text{L},n,k}^{-}\right)\\ \vdots \\ h_{M}\left({\hat{\text{X}}}_{\text{L},n,k}^{-}\right) \end{array}\right]=\\ =\left[\begin{array}{c} \sqrt{{\left({\hat{x}}_{\text{L},n,k}^{-}-xb_{\text{L},n,2\;}\right)}^{2}+{\left({\hat{y}}_{\text{L},n,k}^{-}-yb_{\text{L},n,2\;}\right)}^{2}+{\left(z_{\text{MR}\;}-zb_{\text{L},n,2\;}\right)}^{2}}\\ -\sqrt{{\left({\hat{x}}_{\text{L},n,k}^{-}-xb_{\text{L},n,1\;}\right)}^{2}+{\left({\hat{y}}_{\text{L},n,k}^{-}-yb_{\text{L},n,1\;}\right)}^{2}+{\left(z_{\text{MR}\;}-zb_{\text{L},n,1\;}\right)}^{2}}\\ \vdots \\ \sqrt{{\left({\hat{x}}_{\text{L},n,k}^{-}-xb_{\text{L},n,M\;}\right)}^{2}+{\left({\hat{y}}_{\text{L},n,k}^{-}-yb_{\text{L},n,M\;}\right)}^{2}+{\left(z_{\text{MR}\;}-zb_{\text{L},n,M\;}\right)}^{2}}\\ -\sqrt{{\left({\hat{x}}_{\text{L},n,k}^{-}-xb_{\text{L},n,1\;}\right)}^{2}+{\left({\hat{y}}_{\text{L},n,k}^{-}-yb_{\text{L},n,1\;}\right)}^{2}+{\left(z_{\text{MR}\;}-zb_{\text{L},n,1\;}\right)}^{2}} \end{array}\right] \end{array}$$

For the spherical case, the observation noise is Gaussian and uncorrelated Equation (39):

(39) $${\text{R}}_{\text{L},n}=\left[\begin{array}{ccc} \sigma_{w_{\text{L},n}}^{2} & 0 & 0\\ 0 & \ddots & 0\\ 0 & 0 & \sigma_{w_{\text{L},n}}^{2} \end{array}\right]$$

In the hyperbolic case, the noise is Gaussian, but correlated, and it can be simplified as indicated in Expression (40) according to [17]:

(40) $${\text{R}}_{\text{L},n}=\left[\begin{array}{ccc} \sigma_{w_{\text{L},n}}^{2} & 0.5\cdot \sigma_{w_{\text{L},n}}^{2} & 0.5\cdot \sigma_{w_{\text{L},n}}^{2}\\ 0.5\cdot \sigma_{w_{\text{L},n}}^{2} & \ddots & 0.5\cdot \sigma_{w_{\text{L},n}}^{2}\\ 0.5\cdot \sigma_{w_{\text{L},n}}^{2} & 0.5\cdot \sigma_{w_{\text{L},n}}^{2} & \sigma_{w_{\text{L},n}}^{2} \end{array}\right]$$

### 3.5. Global Navigation in a LRLPS Area (Line 20 of Algorithm 1)

One of the major challenges of this algorithm is to solve the case in which the MR is navigating inside LRLPS areas and the position of the MR is required according to the global reference system. In this case, the global vector Equation (43) is updated using the increments of distance and angle between the current point (*k*) Equation (41) and the previous point (*k*−1) Equation (42) of the local state vector. In the case of having the coverage of several LRLPSs, the global state vector is updated considering only the one which has the least trace of its covariance matrix.

(41) $$\Delta {\hat{d}}_{{\text{L}}_{\left|n=\min\text{trace}(P_{\text{L},n,k}\right)}}=\sqrt{{\left({\hat{x}}_{\text{L},n,k\;}-x_{\text{L},n,k-1\;}\right)}^{2}+{\left({\hat{y}}_{\text{L},n,k\;}-y_{\text{L},n,k-1\;}\right)}^{2}}$$

(42) $$\Delta {\hat{\theta }}_{{\text{L}}_{\left|n=\min\text{trace}(P_{\text{L},n,k}\right)}}=\sqrt{\left({\hat{\theta }}_{\text{L},n,k\;}-{\hat{\theta }}_{\text{L},n,k-1\;}\right)}$$

(43) $${\hat{\text{X}}}_{\text{G},k}=\left[\begin{array}{c} {\hat{x}}_{\text{G},k-1}+\Delta {\hat{d}}_{{\text{L}}_{\left|n=\min\text{trace}(P_{\text{L},n,k}\right)}}\cdot \cos\left({\hat{\theta }}_{\text{G},k-1\;}+\Delta {\hat{\theta }}_{{\text{L}}_{\left|n=\min\text{trace}(P_{\text{L},n,k}\right)}}\right)\\ {\hat{y}}_{\text{G},k-1}+\Delta {\hat{d}}_{{\text{L}}_{\left|n=\min\text{trace}(P_{\text{L},n,k}\right)}}\cdot \sin\left({\hat{\theta }}_{\text{G},k-1\;}+\Delta {\hat{\theta }}_{{\text{L}}_{\left|n=\min\text{trace}(P_{\text{L},n,k}\right)}}\right)\\ {\hat{\theta }}_{\text{G},k-1\;}+\Delta {\hat{\theta }}_{{\text{L}}_{\left|n=\min\mathrm{trace}(P_{\text{L},n,k}\right)}} \end{array}\right]$$

**Figure 3.**
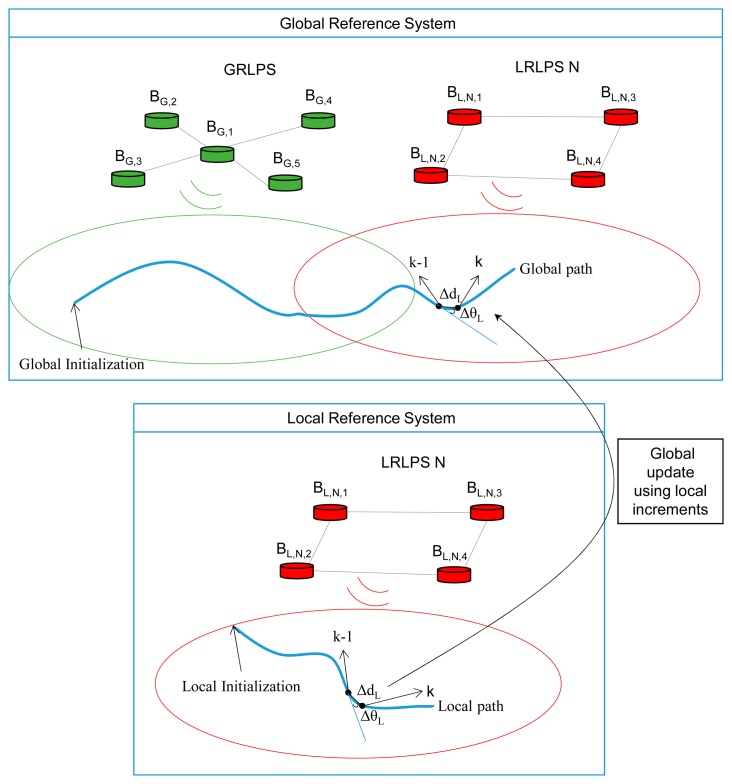
Diagram that shows the update of the global position from the increments of distance and angle between two consecutive instants of time in the local reference system.

Figure 3 shows graphically how the global positioning (related to the global reference system) is performed from the local positioning (obtained regarding the local reference system of an LRLPS). The filter that computes the local positioning algorithm gives positions that are translated and rotated regarding to the global reference system, although the increments of distance and angle between two successive instants of time can be translated from the local path to the global one.

### 3.6. Global Navigation with Only Odometry (Lines 22 and 23 in Algorithm 1)

Of course, when the MR is navigating without any coverage from an LRLPS or a GRLPS, the global state vector is updated using only the odometry information, and the covariance matrix is equal to the *a priori* estimation, as was shown in Equation (13).

Finally, in the cases in which the robot is covered partly by GRLPS and partly by LRLPS, the global state vector is updated using the global beacons. In parallel, the local state vector for the particular LRLPS is updated using the measurements from the local beacons (to be used just in case of losing the global coverage).

## 4. Simulated Results

In order to evaluate the performance of the algorithm, different trajectories have been simulated using the workspace shown in Figure 2. We have compared the proposed algorithm based on GRLPS and LRLPS to a case in which all LPSs were previously calibrated. Results are simulated for both the spherical and the hyperbolic cases.

In the first test, we simulated a trajectory where the MR follows a rectangular path. In this simulation, we have introduced Gaussian noises for the odometry and for the measurement of distances. The typical deviation of the odometry error for the MR increment of distance has been set to zero, while for the increment of angle, we have used a relatively high value (compared with real systems) in order to evaluate the effect of errors when the MR rotates in the corners of the rectangular path. Finally, for distance measurements, we have used a typical error of 1 cm, obtained from real measurements in an Ultrasonic Local Positioning System (ULPS), as seen in Equation (44):

(44) $$\begin{array}{ccc} \sigma_{\Delta d}=0\text{m}, & \sigma_{\Delta \theta }=0.07\text{rad}, & \sigma_{w_{\text{G}}}=\sigma_{w_{\text{L},n}} \end{array}=0.01\text{m}$$

**Figure 4.**
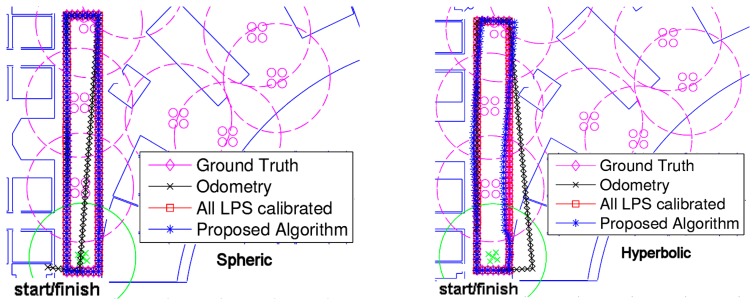
Evaluation of the algorithm for a rectangular trajectory. (**Left**) Spherical case; (**right**) hyperbolic case.

Figure 4 shows the trajectory with only odometry information (lines in black), the ground truth of the trajectory (lines in magenta), the navigation estimation supposing all beacons previously calibrated (lines in red) and the navigation estimation using the proposed algorithm (lines in blue). Obviously, the navigation errors supposing all LPS calibrated are less than the ones using the proposed algorithm, but in the last case, better than the case with only odometry information. The performance of each method can be better seen in Figure 4, where the accumulative mean error evolution is shown.

**Figure 5.**
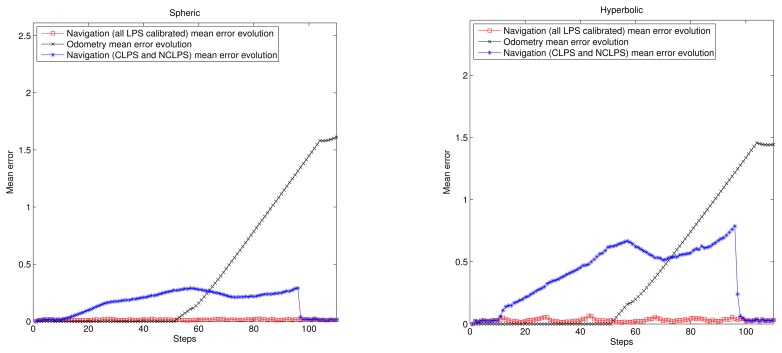
Accumulative mean error evolution. (**Left**) Spherical case; (**right**) hyperbolic case.

In order to evaluate the accumulative error, we have simulated 50 iterations per step of the trajectory assuming the errors described in Equation (44). Note that in Figure 4, only one of these iterations was represented. After all of the iterations, the simulation results are shown in Figure 5, where Step 1 corresponds to the starting point and Step 110 to the finishing point. It can be observed that the navigation errors assuming all LPS calibrated are close to zero and are not accumulative. The odometry has an accumulative error that gets higher right after the first turning of the MR (it is very sensitive to angle errors). Concerning the proposed algorithm, it has a relatively low accumulative error, even when the MR navigates inside LRLPS areas (less than 40 cm for the spherical case and double for the hyperbolic).

Let us pay attention to the navigation error evolution when the MR returns to GRLPS at the end of the trajectory. This happens roughly around Step 100. The error figures go suddenly to very low values again. This is because of the reduced absolute errors that can be obtained under the GRLPS coverage areas [10].

In the second test, an arbitrary route was defined to be followed by the MR. In this case, the odometry errors have been changed, considering typical errors obtained for our real MR and ultrasonic distance measurements based on empirical tests Equation (45):

(45) $$\begin{array}{ccc} \sigma_{\Delta d}=0.07\;\text{m}, & \sigma_{\Delta \theta }=0.05\;\text{rad}, & \sigma_{w_{\text{G}}}=\sigma_{w_{\text{L},\text{n}}} \end{array}=0.01\;\text{m}$$

The defined trajectory covers almost all of the working area, and it was thought to be a practical route.

**Figure 6.**
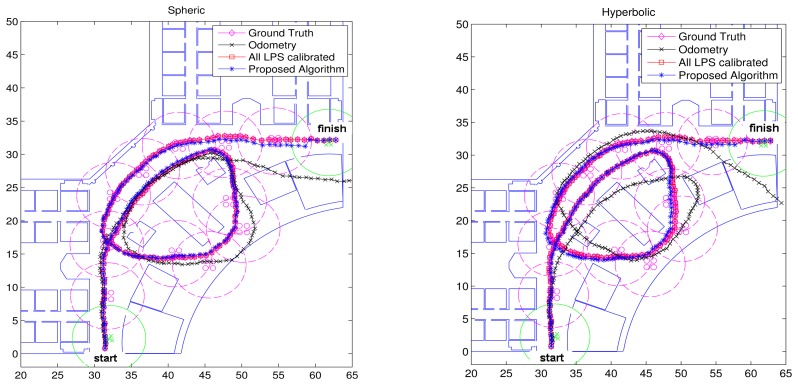
Simulated trajectory. (**Left**) Spherical case; (**right**) hyperbolic case.

Figure 6 shows the results to compare the performance between the proposed algorithm using GRLPS and LRLPS and the case in which all of the LPSs were calibrated, as well as the case with only odometry.

**Figure 7.**
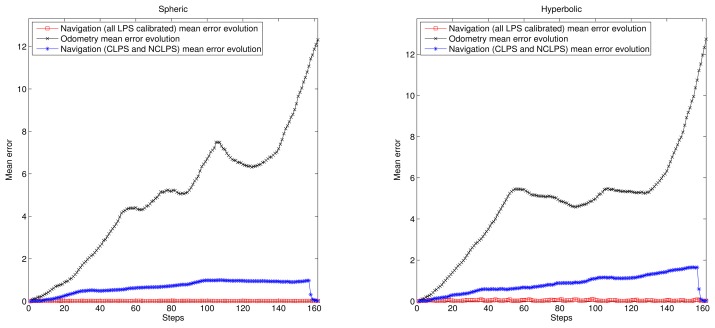
Accumulative mean error evolution for trajectories in Figure 6. (**Left**) Spherical case; (**right**) hyperbolic case.

**Figure 8.**
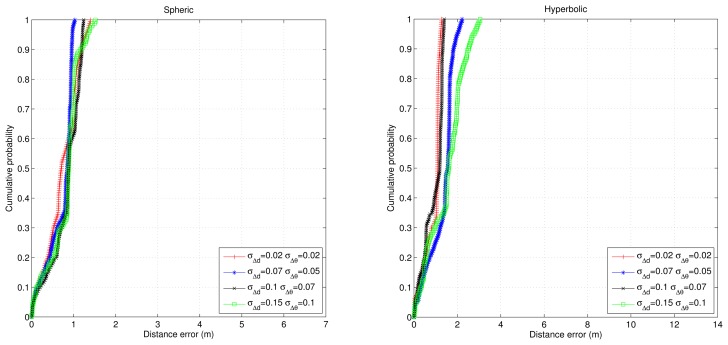
cdf of the navigation errors for different odometry error conditions assuming that the ultrasonic distance errors remain constant. (**Left**) Spherical case; (**right**) hyperbolic case.

**Figure 9.**
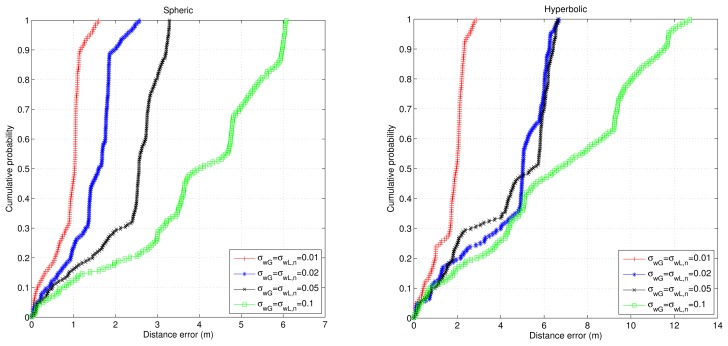
cdf of the navigation errors for different ultrasonic distance error conditions assuming that the odometry errors remain constant. (**Left**) Spherical case; (**right**) hyperbolic case.

Figure 7 shows the accumulative error for the trajectory in this simulation. Now, the trajectory is longer than in the previous simulation, and the accumulative error for the case of using only odometry increases until more than ten meters; while for the proposed algorithms, it remains below 1 m (and better for the spherical case). Finally, Figures 8 and 9 show the cumulative distribution function (cdf) in different error conditions.

For the first test, it can be observed that the variation of odometry errors does not degrade the system performance very much when the ultrasonic distance errors remain constant (Figure 7), whereas when the odometry errors are constant and the ultrasonic distance errors change, the navigation errors increase considerably, especially in the hyperbolic case. Note that in any case, the results for the hyperbolic trilateration show twice the errors that are obtained using the spherical trilateration.

## 5. Real Results

**Figure 10.**
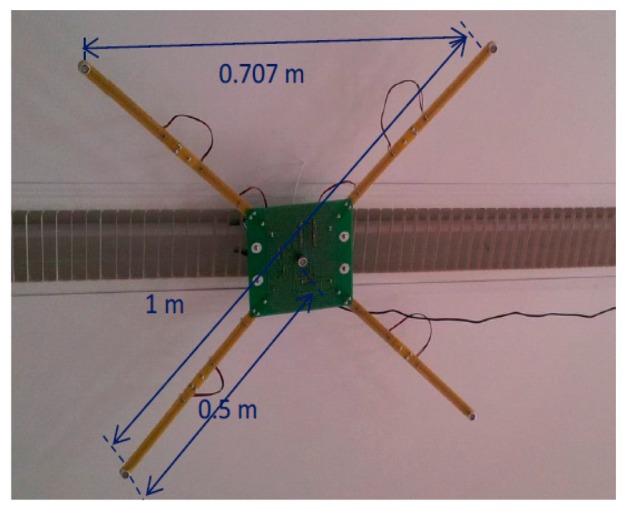
Structure of a real ULPS.

In order to evaluate the algorithm behavior in a real scenario, we have used two ultrasonic LPSs. One of them will work as a GRLPS, while the other will operate as an LRPLS. The layout and hardware of both LPSs are the same. Each LPS has the structure shown in Figure 10.

There are five emitters (beacons) placed, four of them on the corners of a square and the fifth one in its center. All of them emit a 1023-bit Kasami code in order to increase the detection performance. Full details of the system are explained in [10].

**Figure 11.**
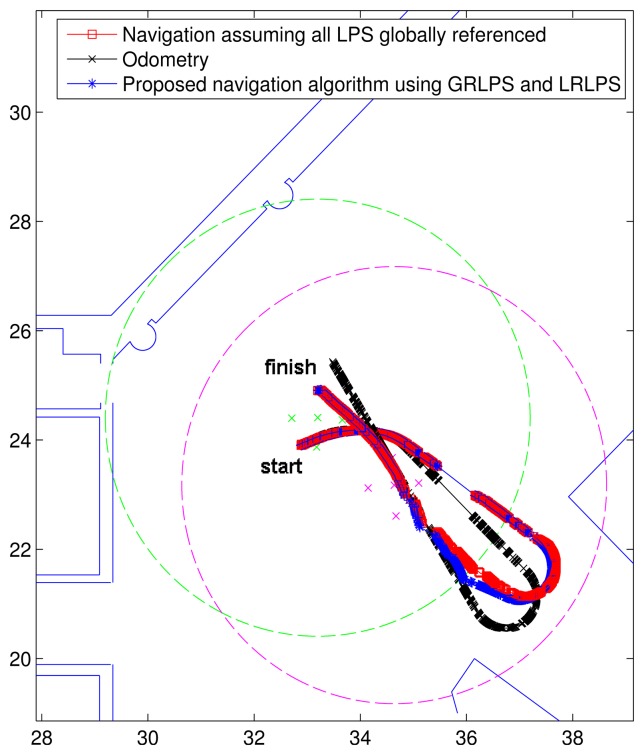
Real results with a GRLPS and an LRLPS.

Figure 11 shows the test trajectory performed with a Pioneer 3-DX MR [18]. The coverage area of the GRLPS is shown in green, while the coverage area of the LRLPS is shown in magenta. To estimate the MR location, it is only possible to use the hyperbolic case, since there is no synchronization between the beacons and the receiver.

In this Figure 11, the red line (with squares) is the estimated path assuming all LPSs were globally referenced. As the position errors in this case are close to the ground truth, this path will be used as the reference one to be compared with the results obtained in other conditions. The black line (with crossings) represents the trajectory described by the odometry of the robot, while the blue line (with asterisks) illustrates the results obtained using our proposed algorithm. It can be observed that using this algorithm, the estimated trajectory is close to the one assuming that all LPS were calibrated.

## 6. Use of the System for Human Tracking

The approach presented in this paper is suitable for human navigation, provided that the equipment is conveniently adapted.

Regarding the US reception stage, the MR uses a receiver system developed by Avisoft [19] connected to a laptop. However, this receiver is too huge to be carried by a person, and thus, in [20] we proposed an ultrasonic signal acquisition module for mobile devices (smartphones, tablets).

Regarding the odometry data obtained from people walking, different approaches have been published. For instance, taking advantage of the smartphone sensors, some authors, as in [21], propose a pedestrian dead reckoning technique based on the accelerometer sensor, which permits the recognition of some step patterns. On the other hand, Inertial Measurement Units (IMUs) allow getting better estimations than smartphones; although the equipment required is more intrusive. Each IMU is usually composed of three accelerometers and tree gyroscopes and is mounted on the foot. An example of this approach can be found in [22].

**Figure 12.**
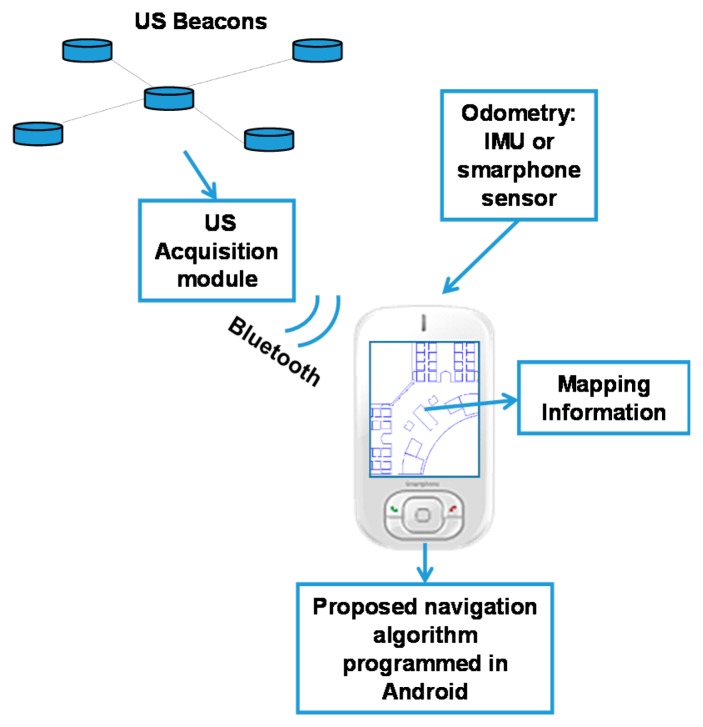
Proposed scheme for human tracking.

As future work, we intend to use the ultrasonic signal acquisition module and a pedestrian dead reckoning method in order to apply the navigation algorithm presented in this paper for human tracking and navigation. A major challenge will be the adaptation of our algorithm to a smartphone due to real-time restrictions. Figure 12 shows a scheme of the adaptation for human tracking.

## 7. Conclusions

In this paper, we have proposed a novel algorithm that allows navigating with increased performance inside an extensive area covered by a set of LPSs, some of them with a global reference (GRLPSs) and most with a local reference system (LRLPSs). The main advantage of the proposed algorithm is the possibility of navigating in extensive indoor environments without calibration of all of the needed LPSs, useful in cases of changing environments or when a fast installation is required.

In the case that the MR navigates inside an LRLPS coverage area, the accumulative position error increases slowly (compared with the case of only using odometry), and usually, it can be admissible for MR indoor navigation; this error is reset (decreases to a low value) when the MR is inside a GRLPS coverage area. The performance of the system has been tested with different simulated and real trajectories. Finally, we have studied the possibility to adapt the system to human tracking as future work.
